# Sequencing and characterization of leaf transcriptomes of six diploid *Nicotiana* species

**DOI:** 10.1186/s40709-016-0048-5

**Published:** 2016-04-18

**Authors:** Ni Long, Xueliang Ren, Zhidan Xiang, Wenting Wan, Yang Dong

**Affiliations:** Faculty of Life Science and Technology, Kunming University of Science and Technology, South Jingming Road No.727, Kunming, 650500 Yunnan China; Guizhou Tobacco Research Institute, North Yuntan Road, Jinyang District, Guiyang, 550003 Guizhou China; State Key Laboratory of Genetic Resources and Evolution, Kunming Institute of Zoology, Chinese Academy of Sciences, 32 East Jiaochang Road, Kunming, 650223 Yunnan China; Biological Big Data College, Yunnan Agricultural University, Kunming, 650201 Yunnan China

**Keywords:** *Nicotiana*, Transcriptome, De novo assembly, Phylogenetic relationship, *Nicotiana setchellii*, *Nicotiana cordifolia*, *Nicotiana knightiana*, *Nicotiana tomentosiformis*, *Nicotiana noctiflora*, *Nicotiana glauca*

## Abstract

**Background:**

*Nicotiana* belongs to the Solanaceae family that includes important crops such as tomato, potato, eggplant, and pepper. *Nicotiana* species are of worldwide economic importance and are important model plants for scientific research. Here we present the comparative analysis of the transcriptomes of six wild diploid *Nicotiana* species. Wild relatives provide an excellent study system for the analysis of the genetic basis for various traits, especially disease resistance.

**Results:**

Whole transcriptome sequencing (RNA-seq) was performed for leaves of six diploid *Nicotiana* species, i.e. *Nicotiana glauca*, *Nicotiana noctiflora*, *Nicotiana cordifolia*, *Nicotiana knightiana*, *Nicotiana setchellii* and *Nicotiana tomentosiformis*. For each species, 9.0–22.3 Gb high-quality clean data were generated, and 67,073–182,046 transcripts were assembled with lengths greater than 100 bp. Over 90 % of the ORFs in each species had significant similarity with proteins in the NCBI non-redundant protein sequence (NR) database. A total of 2491 homologs were identified and used to construct a phylogenetic tree from the respective transcriptomes in *Nicotiana.* Bioinformatic analysis identified resistance gene analogs, major transcription factor families, and alkaloid transporter genes linked to plant defense.

**Conclusions:**

This is the first report on the leaf transcriptomes of six wild *Nicotiana* species by Illumina paired-end sequencing and de novo assembly without a reference genome. These sequence resources hopefully will provide an opportunity for identifying genes involved in plant defense and several important quality traits in wild *Nicotiana* and will accelerate functional genomic studies and genetic improvement efforts of *Nicotiana* or other important Solanaceae crops in the future.

**Electronic supplementary material:**

The online version of this article (doi:10.1186/s40709-016-0048-5) contains supplementary material, which is available to authorized users.

## Background

The genus *Nicotiana* is a member of the Solanaceae or nightshade family, which includes many economically important crop plants such as tomato, potato, eggplant, and pepper. According to Goodspeed [1] and Goodspeed & Thompson [2], *Nicotiana* was initially divided into three subgenera and 14 sections. Recently, this genus was reclassified into 13 sections based on morphological, cytological, and DNA sequence data [3, 4]. *Nicotiana* includes over 75 naturally occurring species, almost half of which are allopolyploid [3]. The genus *Nicotiana* contains species of scientific and economic importance, with different evolutionary histories resulting to highly complex genomes [5]. Of all species, only *Nicotiana tabacum* (common tobacco) and *Nicotiana rustica* are cultivated worldwide, whereas the others are wild species. Moreover, *Nicotiana benthamiana* is used extensively as a model to study plant-pathogen interactions. Several other species, such as *Nicotiana alata* and *Nicotiana sylvestris,* are grown as ornamentals. In *N. tabacum* breeding programs, wild *Nicotiana* species are valuable sources for identifying genes involved in disease and pest resistance, important quality traits, and phytochemicals, which are not present in cultivated varieties [6].

Plants are constantly under the attack of bacteria, fungi, viruses, nematodes and insect pests. Some of them have successfully invaded crop plants, causing diseases and reducing crop quality and yield. To protect against pathogens, plants have evolved various defense mechanisms. Plant disease resistance (R) genes play a key role in defending plants from a range of pathogens. For instance, N genes from tobacco confer resistance to tobacco mosaic virus (TMV) [7]. In recent years, a set of 112 known and 104,310 putative R genes fighting against 122 different pathogens have been identified in 233 plant species [8]. Most of the characterized R genes share a few highly conserved domains, including nucleotide binding site (NBS), leucine-rich repeat (LRR), Toll/Interleukin-1 receptor (TIR) and coiled-coil (CC) domains [9–11]. These conservative domains provide convenient and reliable means for rapidly identifying and cloning R genes or resistance gene analogs (RGAs).

Identification of *Nicotiana* R genes and RGAs cannot only help elucidate the molecular mechanisms of host-pathogen interaction, but also benefit breeding programs for disease resistance in *Nicotiana* and other important Solanaceae crops. Transcriptomic sequences can be useful substitutes for gene discovery in species without sequenced genomes. In the past, a large RGA pool has been mined from transcriptomic sequences and expressed sequence tags (ESTs) of coffee [12], *Phaseolus vulgaris* [13], *Curcuma longa* [14] and *Cocos nucifera* [15]. Wild *Nicotiana* species are known to resist a variety of pathogens. For example, *N. glauca* has attractive potentials to resist black root rot (BRR), potato virus Y (PVY), tobacco etch virus (TEV), anthracnose (An), powdery mildew (PM), rattle virus (RV) and tobacco streak virus (TS) [16–18]. *Nicotiana noctiflora* is resistant to PM and PVY. *Nicotiana cordifolia* shows resistance to TS. *Nicotiana knightiana* manifests high resistance to An, PM, root knot nematodes (RK), PVY and TEV. *Nicotiana setchellii* shows resistance to RV and TEV. *Nicotiana tomentosiformi*s is resistant to cyst nematodes (CN), RK, RV and TEV [16, 17]. These observations suggest that wild *Nicotiana* species are excellent depositories of R genes and RGAs, but relevant analyses of these genes have been lacking.

In *Nicotiana* species, alkaloids (e.g. nicotine) are believed to function as a chemical defense mechanism against pathogens and herbivores. Nicotine and related pyridine alkaloids are synthesized in the tobacco root and then translocated to the aerial parts of the plant [19, 20]. Thus the translocation of nicotine from the root to the leaves is very important in tobacco defenses.

Comparative studies of closely related species can advance our understanding of the genetic architecture of adaptive traits. So far, such studies have been very limited for several crops including tobacco. This is mainly due to the lack of genomic resources hampering the development of genetic markers for investigating species divergence, adaptation and demographic processes in natural populations.

In the present study, we selected six wild *Nicotiana* species for analyses, which included *N. glauca, N. noctiflora, N. cordifolia, N. knightiana, N. setchellii,* and *N. tomentosiformis*. These diploid *Nicotiana* species (all with chromosome numbers of 2n = 24) were chosen because they are repositories of pathogen resistant genes (Table 1). The six wild *Nicotiana* species belong to three sections: *Noctiflorae*, *Paniculatae* and *Tomentosae*. Trait introgression from wild relatives has been used to improve crop species. For example, characters from at least 13 different species have been transferred into tobacco [4]. With advances in next-generation sequencing (NGS) technologies, genomic data for several *Nicotiana* species have become available [21–25]. These data revealed that some *Nicotiana* genomes are large compared with other Solanaceae species such as the tomato [5]. For most wild *Nicotiana* species, very few genomic sequences are currently available. In this study, we performed transcriptome sequencing using the Illumina paired-end sequencing technique with the aim of identifying expressed RGAs, transcription factors important in plant defense, and alkaloid transporter genes by data mining. Our results will provide a useful basis for future identification and cloning of interest genes in wild *Nicotiana* and contribute to the improvement of cultivated tobacco and other important Solanaceae crops.

**Table 1:** Summary of the six wild *Nicotiana* species investigated in this study

Species	Sections	Subgenus	Resistance to diseases
*N. glauca*	*Noctiflorae*	*Petunioides*	BRR, An, PM, RV, TEV, TS, PVY
*N. noctiflora*	*Noctiflorae*	*Petunioides*	PM, PVY
*N. cordifolia*	*Paniculatae*	*Rustica*	TS
*N. knightiana*	*Paniculatae*	*Rustica*	An, PM, RK, TEV, PVY
*N. setchellii*	*Tomentosae*	*Tabacum*	RV, TEV
*N. tomentosiformis*	*Tomentosae*	*Tabacum*	CN, RK, RV, TEV

*BRR* black root rot, *An* anthracnose, *CN* cyst nematodes, *PM* powdery mildew, *RK* root-knot nematodes, *RV* rattle virus, *TS* tobacco streak virus, *PVY* potato virus Y, *TEV* tobacco etch virus

## Results and discussion

### Assembly of RNA-seq reads and evaluation

The Illumina paired-end sequencing yielded 100 bp paired-end independent reads from each insert of cDNA. After stringent quality assessment and data filtering, reads with Q20 bases (those with a base quality greater than 20) were selected as high quality reads for further analysis. In this study, 9.0–22.3 Gb of clean data were generated for each sample (Additional file 1). Due to the lack of reference genome information, Trinity was used for de novo assembly of the six wild *Nicotiana* species [26]. We ultimately obtained 182,046, 146,188, 134,519, 67,073, 102,935 and 117,640 transcripts with length >100 bp for *N. glauca, N. noctiflora, N. cordifolia, N. knightiana, N. setchellii* and *N. tomentosiformis,* respectively (Additional file 1). Subsequently, open reading frames (ORFs) were predicted and the transcripts were translated into peptides culled at a minimum length of 100 amino acids. Only ORFs longer than 300 bp were considered to be possible protein-encoding transcripts and 33,995–79,449 ORFs were obtained through this process for the studied species (see Additional file 2). Although the ORFs of the six wild *Nicotiana* species varied within a large range, from 33,995 to 79,449, after removing redundancy due to alternative splicing isoforms, the ORFs ranged from 22,168 to 29,356 (*N. glauca* 22,934*, N. noctiflora* 26,788*, N. cordifolia* 29,356*, N. knightiana* 22,168*, N. setchellii* 26,579 and *N. tomentosiformis* 24,213).

In the absence of a reference genome, evaluating the quality of the de novo assembled transcriptomes becomes a tedious job. To resolve it, we marked *N. tomentosiformis* as a reference. A total of 53,753 reported peptide sequences (ftp://solgenomics.net/genomes/Nicotiana_tomentosiformis/annotation/, Accessed 27th Apr 2015) were blasted [27] against our predicted ORFs of *N. tomentosiformis* using BLASTp with a cut-off e-value of 10^−5^. A total of 50,390 (93.74 %) *N. tomentosiformis* proteins had a BLAST hit in our ORFs and 32,761 (60.95 %) proteins showed ≥90 % identity with more than 50 % matched length of the corresponding proteins, which suggests our assembly should be largely complete. Moreover, ORFs were compared to the core eukaryote gene (CEG) set of 248 proteins from six reference species [28] to assess the quality of each transcriptome. The CEGs were well-represented in the assembled transcriptomes of the *N. glauca*, *N. noctiflora*, *N. cordifolia*, *N. knightiana*, *N. setchellii*, *N. tomentosiformis*, with significant matches (alignment length ≥50 % CEG length and e-value <10^−5^) to 87.10, 92.34, 91.94, 89.92, 90.73 and 91.53 % of the CEGs, respectively. This indicated that the quality and completeness of our transcriptome assemblies were high enough for subsequent analyses. These transcriptome sequences may greatly enrich the *Nicotiana* sequence database, and will be useful in trait-related gene mining, such as the identification of plant defense genes.

### Transcriptome annotation and expression analysis

To obtain the most informative and complete annotation, ORFs from six species of *Nicotiana* were annotated separately. Sequence similarity searches were conducted against the NCBI NR and Swiss-Prot databases using the BLASTp algorithm with a cutoff e-value of 10^−5^. Using this approach, 94.68–97.43 % ORFs showed homology with sequences in the NR database (Table 2) and 71.06–77.76 % ORFs returned significant matches in the Swiss-Prot database (Table 2). The e-value distribution of the top hits in the Swiss-Prot database showed that 60.78 % of the mapped sequences had a strong homology (smaller than 10^−5^, Additional file 3). The remaining un-annotated ORFs appeared to be either *Nicotiana*-specific genes or homologous genes with unknown functions in other species.

**Table 2:** Summary of functional annotation of predicted ORFs

	*N. glauca*	*N. noctiflora*	*N. cordifolia*	*N. knightiana*	*N. setchellii*	*N. tomentosiformis*
ORF	65,423	63,930	79,449	33,995	52,916	53,121
NR	63,133	63,930	75,226	33,121	50,490	51,207
Percentage	96.50 %	95.88 %	94.68 %	97.43 %	95.42 %	96.40 %
SwissProt	47,743	47,197	56,453	26,434	38,954	40,138
Percentage	72.98 %	73.83 %	71.06 %	77.76 %	73.61 %	75.56 %
KEGG	37,385	37,007	43,884	20,439	29,817	30,858
Percentage	57.14 %	57.89 %	55.24 %	60.12 %	56.35 %	58.09 %
GO	36,508	35,846	43,279	20,370	30,079	27,592
Percentage	55.80 %	56.07 %	54.47 %	59.92 %	56.84 %	51.94 %

**Fig. 1:**
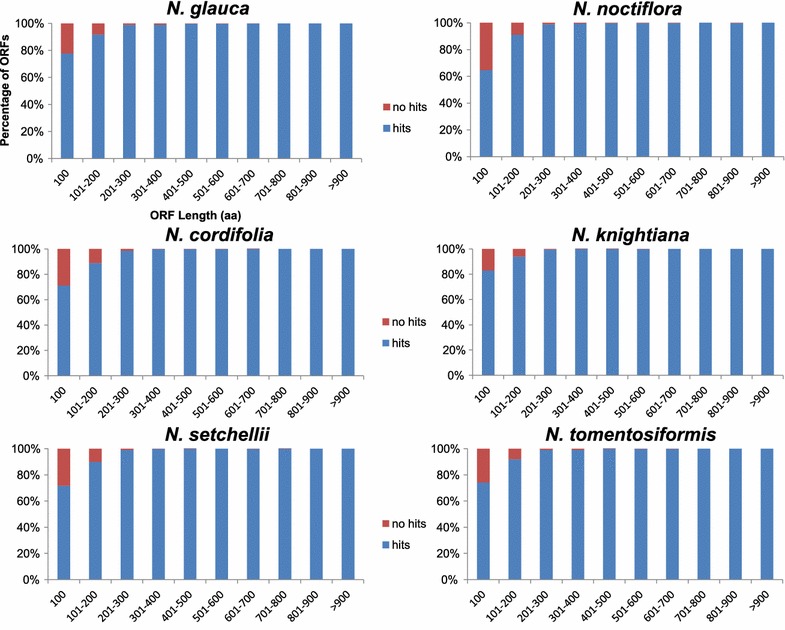
Comparison of ORF length between hit and no hit proteins in NR database. For *N. glauca*, *N. noctiflora*, *N. cordifolia*, *N. knightiana*, *N. setchellii*, *N. tomentosiformis*, longer ORFs were more likely to have BLASTp homologs in protein database

Besides, a higher (>90 %) match rate in the NR database was shown by ORFs with >200 aa in length, whereas ORFs shorter than 200 aa exhibited a lower match rate (Fig. 1). An almost similar match rate pattern was observed in the annotation for Swiss-Prot database (Additional file 4).

The expression level of each ORF from six wild *Nicotiana* species was normalized and quantified by the FPKM (fragments per kilobase per million sequenced reads) method (Additional file 5). The ORFs with FPKM <1 were considered to be unexpressed, ORFs with FPKM values between 1 and 3 were considered lowly expressed, those between 3 and 15 were considered expressed at medium levels, and those with FPKM values >60 were considered highly expressed (Table 3). The top 20 ORFs with highest FPKM values for each species can be seen in Additional file 6. These ORFs either encode chloroplast proteins or play role in photosynthesis. These results are consistent with the fact that leaves are the plant’s main photosynthetic organs.

**Table 3:** Distribution of ORF expressions in six wild *Nicotiana* species

FPKM interval	*N. glauca*	*N. noctiflora*	*N. cordifolia*	*N. knightiana*	*N. setchellii*	*N. tomentosiformis*
0–1	31,857 (48.69 %)	28,985 (45.34 %)	33,698 (42.41 %)	6245 (18.37 %)	16,346 (30.89 %)	18,670 (35.15 %)
1–3	11,832 (18.09 %)	11,105 (17.37 %)	16,828 (21.18 %)	2936 (8.63 %)	11,577 (21.88 %)	11,338 (21.34 %)
3–15	11,696 (17.88 %)	12,299 (19.24 %)	16,812 (21.16 %)	14,986 (44.08 %)	13,912 (26.29 %)	12,948 (24.37 %)
15–60	6710 (10.26 %)	7708 (12.06 %)	8232 (10.36 %)	6782 (19.95 %)	7495 (14.16 %)	6836 (12.87 %)
>60	3328 (5.09 %)	3833 (5.10 %)	3879 (4.88 %)	3046 (8.96 %)	3586 (6.78 %)	3329 (6.27 %)

Ratios of ORF number to total ORF number are presented in parentheses

*FPKM* fragments per kilobase per million sequenced reads

### Phylogenetic analysis

**Fig. 2:**
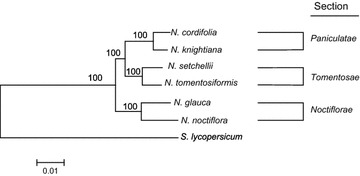
Phylogenetic tree based on the transcriptomes of the six wild *Nicotiana* species and *S. lycopersicum*. Phylogenetic tree was constructed using the neighbor-joining method with 1000 bootstraps. Bootstrap support is shown at the nodes

Large-scale transcriptome data are a potential source of information for multigene phylogenetic analysis (the phylogenomic approach). In this study, 2491 single copy orthologs were identified and two phylogenetic trees were constructed by the neighbor-joining (NJ) method in Phylip [29] (Fig. 2) and maximum likelihood (ML) method in PhyML [30] (Additional file 7). The two phylogenies showed identical topologies. Earlier, Goodspeed placed *N. glauca* in the section *Paniculatae* based on evidence from morphology, cytology, biogeography, and crossing experiments [31]. Later, *N. glauca* was placed in the section *Noctiflorae* based on analysis of sequences from internal transcribed spacer (ITS) of nuclear ribosomal DNA (nrDNA) [3, 32]. Current phylogenetic analysis of the transcriptomes from six diploid species of *Nicotiana* with *Solanum lycopersicum* (tomato) as an outgroup supported the *Noctiflorae*, *Paniculatae,* and *Tomentoase* clades. The phylogenetic trees obtained in the current study placed *N. glauca* in the section *Noctiflorae*, supporting the results of previous works by Chase et al. [32]. and Knapp et al. [3].

### Functional classification by KEGG

**Fig. 3:**
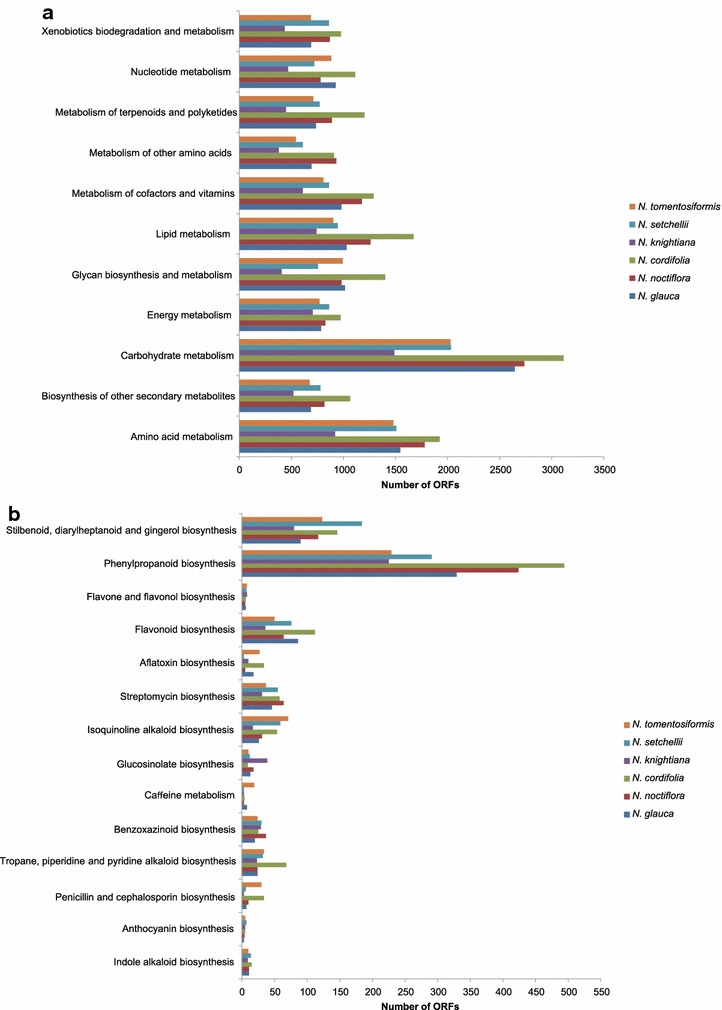
Pathway assignment based on KEGG from the six wild *Nicotiana* species. **a** Classification based on metabolism categories; **b** classification based on secondary metabolism categories

ORFs of six wild *Nicotiana* species were compared with KEGG (Kyoto Encyclopedia of Genes and Genomes) database using BLASTp with an e-value less than 10^−5^, and the corresponding pathways were established. For the six species, 55.24–60.12 % of ORFs were successfully annotated to KEGG pathways (Table 2). Genes within the same pathway usually cooperate with each other to exercise their biological function, and hence pathway-based analysis contributes to the exploration of biological functions and interactions of genes [33]. The sequence annotation in KEGG largely contained metabolic pathways of major biomolecules such as carbohydrates, amino acids, lipids, nucleotides, etc. (Fig. 3a). The metabolic pathways with most representation by proteins were those of carbohydrate metabolism and amino acid metabolism. In the secondary metabolism, for *N. glauca, N. noctiflora, N. cordifolia, N. knightiana, N. setchellii, N. tomentosiformis,* 687, 817, 1064, 518, 779 and 677 proteins were classified into 14 subcategories, respectively (Fig. 3b). Among them, the cluster for “Phenylpropanoid biosynthesis” represents the largest group followed by “Stilbenoid, diarylheptanoid and gingerol biosynthesis”. The phenypropanoid pathway is often considered to be involved in plant resistance [34]. Flavonoids and glucosinolates are secondary metabolites that play important roles in protecting plants against pathogens. We also found unigenes involved in the biosynthesis of flavonoid and glucosinolate. The results will facilitate the discovery of novel genes involved in the specific metabolic pathways and secondary metabolic pathways and will provide a valuable resource for investigating the defense-related pathways in *Nicotiana* and other Solanaceae species.

### Functional classification by GO

**Fig. 4:**
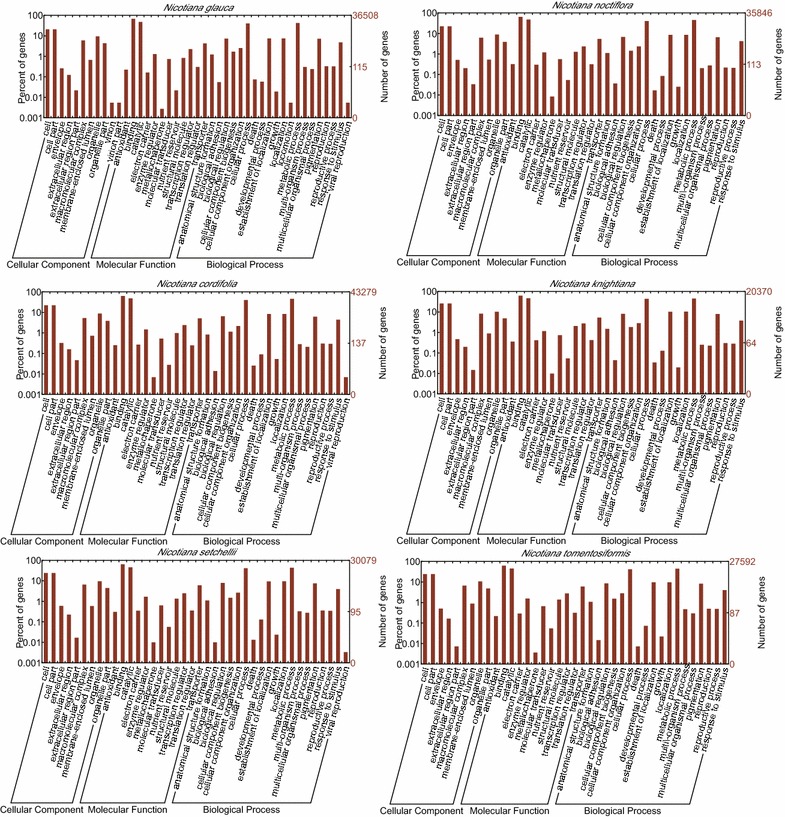
Histogram presentation of GO classification. The GO annotation results from the ORFs of six *Nicotiana* species are summarized in three main categories: biological process, cellular component and molecular function. The right *y-axis* indicates the number of genes in a category. The left *y-axis* indicates the percentage of a specific category of genes in that main category

Gene ontology (GO) [35] provides ontologies of defined terms representing gene product properties and describes gene products in terms of their associated biological processes, cellular components, and molecular functions. In this study, 36,508, 35,846, 43,279, 20,370, 30,079 and 27,592 annotated ORFs corresponding to *N. glauca*, *N. noctiflora*, *N. cordifolia*, *N. knightiana*, *N. setchellii*, and *N. tomentosiformis*, respectively, were assigned to one or more sub-categories of GO terms. The GO terms of the subcategories are presented in Fig. 4. For the six wild *Nicotiana* species, among these groups, genes involved in “metabolic process” and “cellular process” were the most highly represented in the biological process category. Genes involved in other important biological processes such as biological regulation, response to stimulus, and anatomical structure formation process were also identified. Furthermore, a relatively large number of sequences were found to be involved in the metabolism of pigmentation. Within the cellular components category, “cell” and “cell parts” were the most highly represented groups. The molecular function category comprised proteins involved in “binding” and “catalytic activity”. These six wild *Nicotiana* transcriptomes shared broad similarities in the three main categories and many subcategories except viral reproduction.

### Identification of NBS encoding genes and defense response associated transcription factors

The majority of disease resistance genes in plants contain a nucleotide-binding site and leucine-rich repeat (NBS-LRR) domain [36, 37], which confers resistance to fungi, bacteria, viruses, and nematodes. In plants, based on the presence or absence of a TIR homology region at the N-terminus, the NBS-LRR genes can be subdivided into two main groups: TIR-NBS-LRR and non-TIR-NBS-LRR. The latter may have a coiled-coil (CC) motif in the N-terminal region and can be called as CC-NBS-LRR.

To control diseases in certain agriculturally important plants, the identification of resistance genes from their less susceptible relatives has been the top priority in crop breeding programs. In the case of Solanaceae species, the pepper *Bs2* gene with NBS-LRR domain was introduced into tomato lines to develop resistance against bacterial spot disease [38]. In tobacco, the TIR-NBS-LRR encoding N gene was introduced into *N. benthamiana*, which resulted in the acquirement of hypersensitivity response to tobacco mosaic virus (TMV) [39].

In this study, after going through a filtering process, 87–173 unigenes encoding NBS domains were identified from the six wild *Nicotiana* species. These NBS-encoding genes were classified into six classes on the presence or absence of CC domain, TIR domain, and/or LRR domain. These six classes include CC-NBS-LRR, CC-NBS, TIR-NBS-LRR, TIR-NBS, NBS-LRR, and NBS (Table 4). The NBS class was the most represented class (61–113 unigenes) for all six species in the present study. The TIR-NBS class had 3–8 unigenes for each species, and the NBS-LRR class had 5–14 unigenes (0 for *N. tomentosiformis*). Additionally, 2–11 unigenes (0 for *N. cordifolia*) were predicted to encode TIR-NBS-LRR, 11–31 unigenes were identified as CC-NBS, and 3–7 unigenes contained CC-NBS-LRR. The candidate R genes will enhance our knowledge about the mechanisms of disease resistance in Solanaceae species and help breed novel disease resistant varieties.

**Table 4:** Classification of NBS encoding genes based on the predicted domains from six wild *Nicotiana* transcriptomes

	*N. glauca*	*N. noctiflora*	*N. cordifolia*	*N. knightiana*	*N. setchellii*	*N. tomentosiformis*
CC-NBS	28	31	11	12	21	24
CC-NBS-LRR	3	7	2	2	7	5
TIR-NBS	4	4	8	3	5	4
TIR-NBS-LRR	9	11	0	4	2	6
NBS-LRR	14	8	13	5	8	0
NBS	113	112	84	61	84	79
Total	171	173	118	87	127	118

Transcription factors (TFs) are also important in disease resistance. They bind to the promoters of resistance genes and regulate their expression. The TFs related to defense or disease resistance mainly belong to the MYB [40], WRKY [41], bZIP [42] and Whirly [43] families. Overexpression of the defense-related TFs has improved disease resistance in many transgenic crops [44]. By using Pfam annotations, we identified 439–618 candidate unigenes matching the defense-related TFs in the six wild species of *Nicotiana* (Table 5). These candidate TFs will be potential targets for developing the resistant lines of tobacco and other Solanaceae crops.

**Table 5:** Summary of ten transcription factors involved in plant defense in six wild *Nicotiana* transcriptomes

	*N. glauca*		*N. noctiflora*	*N. cordifolia*	*N. knightiana*	*N. setchellii*	*N. tomentosiformis*
MYB		200	221	217	180	225	152
WRKY		48	57	57	50	54	35
ERF-type/AP2-EREBP		78	87	95	67	103	79
CBF		16	15	22	18	20	16
bZIP		55	63	57	45	52	39
SBP/SPL6		20	23	24	20	20	20
NAC domain/NAM		55	65	69	45	58	45
TFIIA		1	2	2	2	2	1
Whirly		3	2	2	2	2	2
Homeo-domain		60	69	73	63	70	50
Total		536	604	618	492	606	439

### Identification of alkaloid transporter genes

Alkaloids are mainly produced in the root and then translocated via xylem transport towards the aerial parts. These toxic chemicals function as part of the chemical defense against invaders [19, 20]. To date, the plant alkaloid transporters are mainly characterized into the ATP-binding cassette (ABC) protein, multidrug and toxic compound extrusion (MATE), and purine permease (PUP) families. Some transporters were found to be required for the efficient biosynthesis of alkaloids in plants [45]. In tobacco, several alkaloid transporter genes have been identified, such as tobacco jasmonate-inducible alkaloid tranporter1 (*Nt*-*JAT1*), *Nt*-*JAT2*, tobacco nicotine uptake permease1 (*Nt*-*NUP1*), *NtMATE1* and *NtMATE2* [46–50].

In the present study, we began our investigation by searching the assembled transcriptome for orthologous genes to known alkaloid transporter genes in the tobacco. *Nt*-*JAT1* transports nicotine and other alkaloids in a proton gradient-dependent manner. *Nt*-*JAT1* mRNA is expressed in leaves, stems, and roots. In leaf cells, *Nt*-*JAT1* localizes to the tonoplast and might play a role in the vacuolar sequestration of nicotine [46]. We found one orthologous gene for *Nt*-*JAT1* in the assembled *N. cordifolia*, *N. setchellii* and *N. tomentosiformis* transcriptomes with high confidence, respectively. For *Nt*-*JAT2* and *NtNUP1* genes, we found orthologous genes in *N. noctiflora*, *N. cordifolia*, *N. knightiana*, *N. setchellii* and *N. tomentosiformis*. According to previous reports, *Nt*-*JAT2* is specifically expressed in leaves. *Nt*-*JAT2* contributes to the transportation of nicotine into the vacuole of leaves [49]. *Nt*-*NUP1* is a plasma membrane-localized nicotine transporter of the PUP family. It is involved in the movement of apoplastic nicotine into the cytoplasm of tobacco root cells, which affects nicotine metabolism and root growth. *NUP1* transcripts are less abundant in the leaves, but are abundant in root tips where nicotine is actively synthesized [48]. Two homologous MATE transporters, *NtMATE1* and *NtMATE2*, were reported to be responsible for the vacuolar accumulation of nicotine in the root. We found one orthologous gene corresponding to the tobacco *MATE1/2* genes in the leaf of *N. noctiflora*. This gene may have a different function.

We did not identify any orthologous genes for *Nt*-*JAT2* and *Nt*-*NUP1* in *N. glauca*. Since *N. glauca* can grow to a tree of several meters tall, it is possible that the acropetal transport of defensive alkaloid is relatively inefficient.

## Conclusions

It is well-known that genetic diversity is essential for the continuous genetic modification and improvement of cultivated crops, as well as for many basic studies in plant biology. As an economic crop, cultivated tobacco has received a fair number of desired genes from wild *Nicotiana* relatives [51–57]. Although wild *Nicotiana* species have played important roles in many research areas of plant biology, their genomic resources have been slowly developed relative to most other major crop species. With this study, we provide the reference transcriptome sequences of six wild *Nicotiana* species for public use. By constructing phylogenetic trees, we confirmed the classification of *Nicotiana* into three sections and the placement of these wild species in each section. Our study will provide a better understanding of the genomic architecture of wild *Nicotiana* and help elucidate genes involved in plant defense. It is likely that these *Nicotiana* species will be used as model systems for investigating many aspects of general plant biology in future. These sequences will be an important resource for evolutionary and developmental genetics in the genus *Nicotiana* and will contribute significantly to the improvement of cultivated tobacco and other important Solanaceae crops.

## Methods

### Plant materials and RNA extraction

Six wild species of *Nicotiana*, including *N. setchellii*, *N. cordifolia*, *N. knightiana*, *N. tomentosiformis*, *N. noctiflora*, and *N. glauca*, were grown in a greenhouse in Guizhou Province under the same cultivation conditions. Fresh leaves from 30-day-old flowerless plants were collected, snap frozen in liquid nitrogen, and stored at −70 °C. RNA was purified using TRIzol (Invitrogen, CA, USA) from the frozen materials according to the manufacturer’s instructions. RNA degradation and contamination was monitored on 1 % agarose gels. RNA integrity was confirmed using the 2100 Bioanalyzer (Agilent Technologies) with a minimum RNA integrated number value of 8 after checking the RNA purity and concentration.

### Library preparation and Illumina sequencing

RNA sequencing libraries were constructed in parallel from the six species using TruSeq RNA Sample Prep Kits (Illumina, SanDiego, USA). Briefly, first strand cDNA synthesis was performed with oligo-dT primer and Superscript II reverse transcriptase (Invitrogen, CA, USA). The second strand was synthesized with *Escherichia coli* DNA Pol I (Invitrogen, CA, USA). Double-stranded cDNA was purified with a Qiaquick PCR purification kit (Qiagen), and sheared with a nebulizer (Invitrogen, CA, USA) into 200–250 bp fragments. After the end repair and addition of a 3′-dA overhang, the cDNA was ligated to Illumina PE adapter oligo mix (Illumina). The products were then purified and enriched with PCR to create the final sequencing cDNA library. Both ends of the library were sequenced on the Illumina HiSeq 2000 platform.

### De novo transcriptome assembly

Before performing the assembly, raw reads (FASTQ format) were cleaned by removing reads containing adaptor sequences, reads containing poly-N, and low-quality reads. For each species, de novo transcriptome assembly was performed using Trinity [26] (version: trinityrnaseq_r2012-06-08) with default settings except min_kmer_cov set to 2, which is a method for the efficient and robust de novo reconstruction of transcriptomes. Afterwards, transcripts with length <200 bp in each species were removed. The protein-coding region prediction program in the Trinity software suite (transcripts_to_best_scoring_ORFs.pl) was used to identify putative open reading frames (ORFs) consisting of at least 100 amino acids on the basis of nucleotide composition.

### Functional annotation and expression level analysis

Peptide annotation for the ORFs obtained in this study was performed by BLASTp (version 2.2.29 +) [27] searching in the NCBI NR database (24th June 2014) and Swiss-Prot database (28th April 2015). An e-value cutoff of 10^−5^ was used and only one best hit was retained for each sequence query. To assign preliminary GO terms to the ORFs, InterProScan (version 5.4) [58] was used to screen the annotated peptide sequences against all the default databases. GO classification of the ORFs was conducted based on biological processes, molecular function, and cellular component and subsequently was visualized by the WEGO online tool [59]. Pathway assignments were carried out based on the KEGG database (8th March 2011) [60]. The ORFs from each species were first compared with KEGG database using BLASTp (version 2.2.29 +) with an e-value less than 10^−5^. An in-house Perl script (https://github.com/NiLong/kegg_stat/) was developed to retrieve KO (KEGG Orthology) information from BLASTp results and correlation between peptides and database pathway was established. The RSEM software (version 1.2.13) [61] was used to quantify the expression level of the ORFs of six wild *Nicotiana* species measured as FPKM values.

### Gene family and phylogenetic analysis

The initial set of annotation contained a high level of redundancy as more than half of the annotated transcripts were alternative splicing isoforms [62]. To avoid this redundancy in subsequent analyses, ORFs from six *Nicotiana* species were clustered using cd-hit-est command in CD-HIT v4.6.1-2012-08-27 [63] with >95 % similarity cutoff and only the representative ORFs in each cluster were retained. This yielded non-redundant sequence datasets for *N. glauca* (22,934 genes), *N. noctiflora* (26,788 genes), *N. cordifolia* (29,356 genes), *N. knightiana* (22,168 genes), *N. setchellii* (26,579 genes) and *N. tomentosiformis* (24,213 genes). These non-redundant sequences were defined as unigenes. Similarly, tomato coding sequences (CDSs) obtained from ITAG2.4 were also clustered and 33,721 genes were obtained. OrthoMCL v1.4 [64] was used to identify ortholog relationships between the six wild *Nicotiana* species and tomato.

Phylogenetic trees were constructed using sequences of 2491 single copy orthologs from six *Nicotiana* species with *S. lycopersicum* (tomato) as an outgroup. Multiple sequence alignments were performed by MUSCLE v3.8.31 [65]. Two methods were used to reconstruct phylogenetic trees: (1) the neighbor-joining method in Phylip v3.696 [29] and bootstrapped with 1000 replicates, and (2) the maximum likelihood method in PhyML v3.0 [30].

### Identification of NBS containing genes and transcription factors related to disease resistance

For the identification of the NBS-encoding genes in this study, we followed the method described for diploid cotton *Gossypium raimondii* [66]. Firstly, the protein sequences of 112 manually curated reference disease resistance genes were collected from the plant resistance gene database (http://www.prgdb.org)  [8]. Non-redundant protein sequences (unigenes) from the six wild *Nicotiana* species were subsequently checked for sequence homology with at least one resistance protein contained in the reference dataset using BLASTp (version 2.2.29+) (scores ≥100 and e-values ≤10^−5^). In a second step, all the BLAST hits were used for further analysis and were screened for protein domains by InterProScan version 5.4 [58]. In the third step, the genes with NBS domain were filtered out according to the NBS domain annotation (PF00931) given by the Pfam database (v27.0) [67]. Subsequently, the Pfam database, SMART protein motif analyses (Simple Modular Architecture Research Tool) (v6.2) [68], and the ncoils program (version 2.2) [69] were used to classify the NBS genes based on TIR, NBS, LRR and CC motifs. The program ncoils was used by InterProScan version 5.4 with default settings to predict coiled-coils domains. Pfam database (v27.0) and SMART protein motif analysis (v6.2) were used to detect the TIR (PF01582) and LRR domains (PF00560, PF07723, PF07725, PF12799, PF13306, PF13504, PF13516, PF13855, and PF14580).

Ten different TF families involved in disease resistance were retrieved from the literature by Sood et al. [70]. Then the unigenes were searched against the domains of ten TFs, including MYB (PF00249, PF13921, PF14379), WRKY (PF03106), ERF-type/AP2-EREBP (PF00847), CBF (PF02312, PF00808, PF03914), bZIP (PF00170, PF03131, PF07716, PF12498), SBP/SPL6 (PF03110), NAC domain/NAM (PF02365, PF14303), TFIIA (PF03153, PF02268, PF02751), Homeo-domain (PF00046, PF05920, PF00157, PF13384, PF13565) and Whirly (PF08536) using the Pfam database from InterProScan version 5.4, respectively. Finally, proteins matching with the Pfam IDs were selected as TFs associated with disease resistance.

### Identification of alkaloid transporter genes

The coding sequences of *Nt*-*JAT1* (accession number AM991692), *Nt*-*JAT2* (accession number AB922128), *NtMATE1* (accession number AB286961), *NtMATE2* (accession number AB286962) and *NtNUP1* (accession number GU174267) of *N. tabacum* were retrieved from NCBI. To identify orthologs of the alkaloid transporter genes in each species, we first performed a bidirectional BLAST search of the alkaloid transporter genes and unigenes from the six species against each other (identity ≥80 %, e-values ≤10^−5^, query coverage ≥50 %).

### Availability of supporting data

RNA-seq data have been deposited in the NCBI sequence Read Archive under the accession numbers SRR2106216, SRR2106514, SRR2106516, SRR2106517, SRR2106530 and SRR2106531. The data set supporting the results of this article is included within the additional files.

## Additional files

10.1186/s40709-016-0048-5 Characteristics of raw data and assembled transcripts in six wild *Nicotiana* species.
10.1186/s40709-016-0048-5 Length distribution of the ORFs predicted from the transcripts of six wild *Nicotiana* species. Length values are represented in amino acids.
10.1186/s40709-016-0048-5 E-value distribution of the top BLAST hits for each ORF in the Swiss-Prot database. The cutoff e-value was set to 10^−5^.
10.1186/s40709-016-0048-5 Comparison of ORF length between hit and no hit proteins in Swiss-Prot database. For *N. glauca, N. noctiflora, N. cordifolia, N. knightiana, N. setchellii, N. tomentosiformis*, longer ORFs were more likely to have BLASTp homologs in protein database.
10.1186/s40709-016-0048-5 The expression levels of ORFs (FPKM) in the six *Nicotiana* species.
10.1186/s40709-016-0048-5 The top 20 abundant ORFs in the six *Nicotiana* species.
10.1186/s40709-016-0048-5 Phylogenetic tree based on the transcriptomes of six *Nicotiana* and *S. lycopersicum* using ML method.
