# Supplementary figures and images for: Sequencing and characterization of leaf transcriptomes of six diploid Nicotiana species
Source: J Biol Res (Thessalon). 2016 Apr 18;23:6. doi: 10.1186/s40709-016-0048-5 (PMC4835900; doi:10.1186/s40709-016-0048-5)

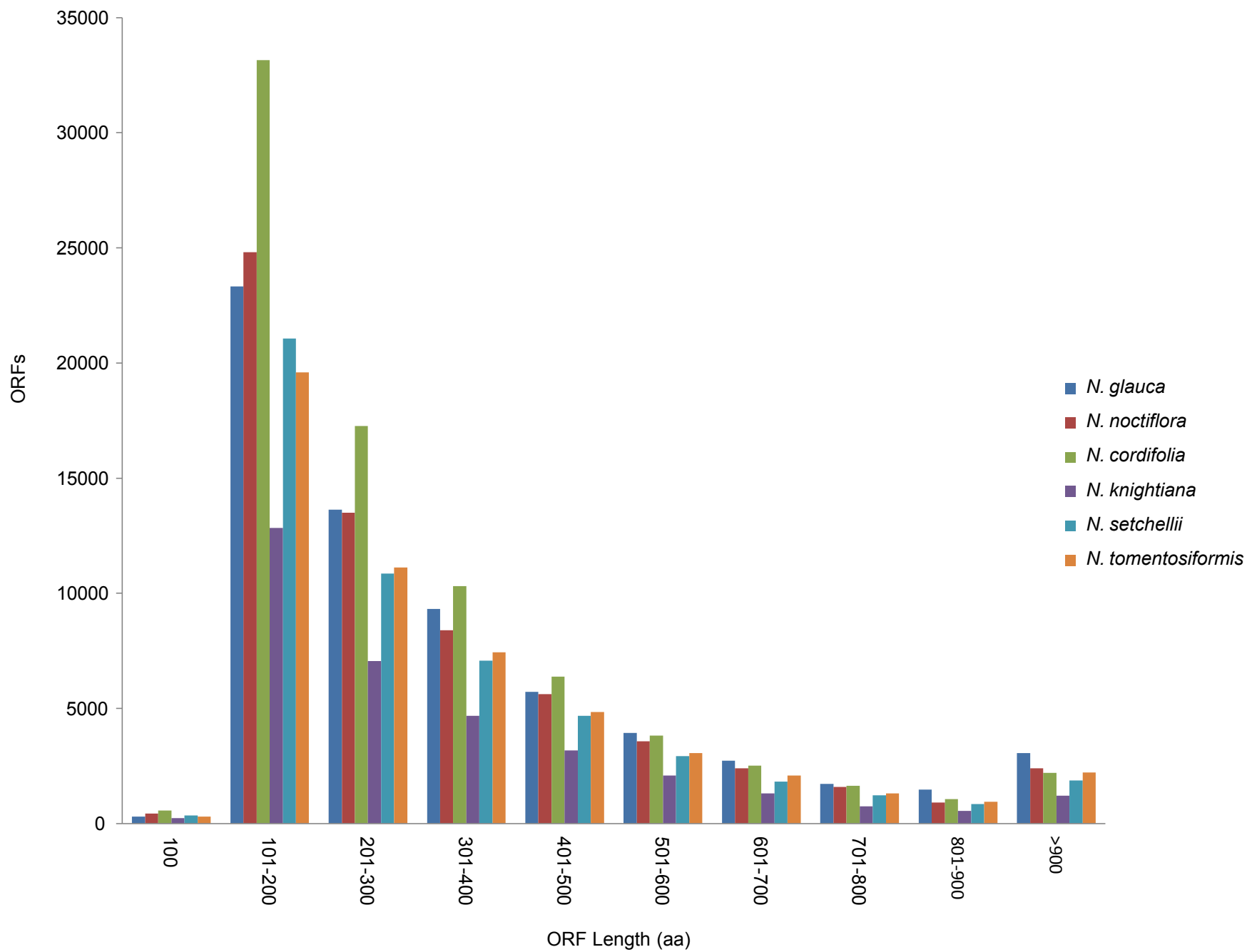

Supplement: Supplementary file 1 — 10.1186/s40709-016-0048-5 Characteristics of raw data and assembled transcripts in six wild Nicotiana species. [file 40709_2016_48_MOESM1_ESM.pdf]

***N. glauca***

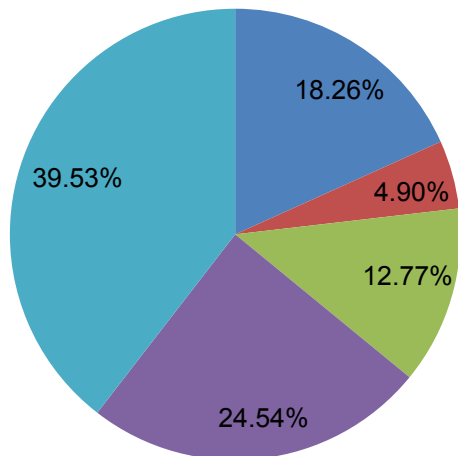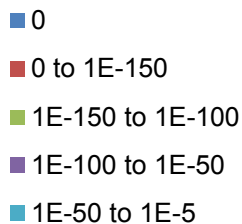

***N. cordifolia***

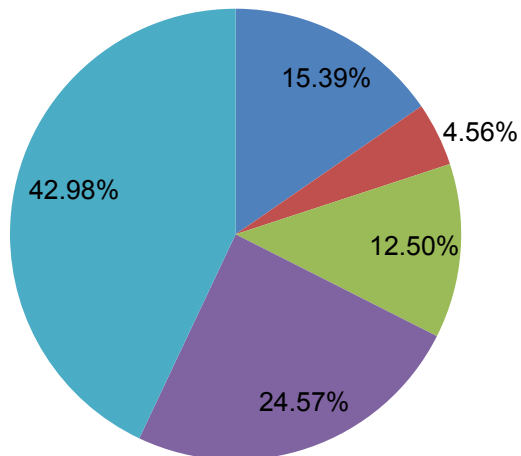

***N. noctiflora***

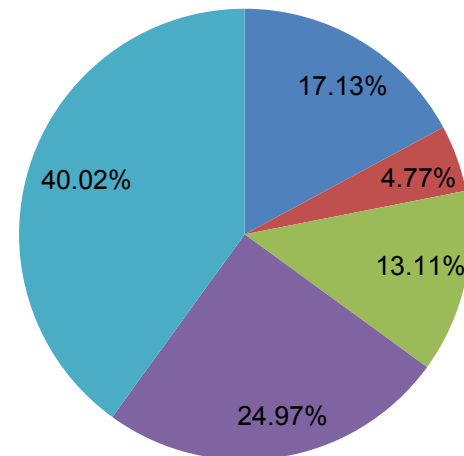

***N. knightiana***

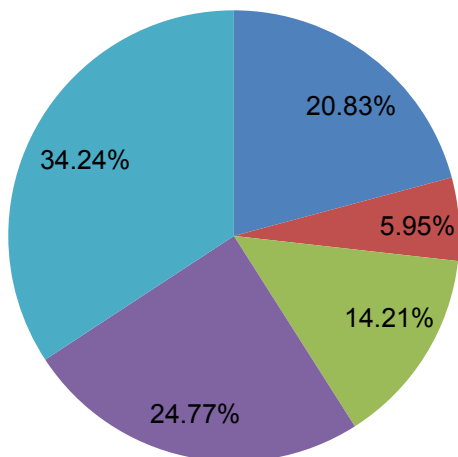

***N. setchellii***

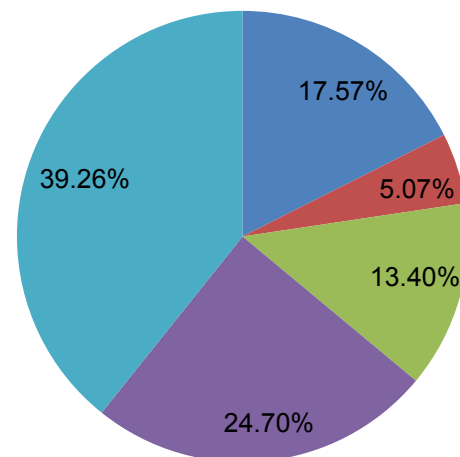

***N. tomentosiformis***

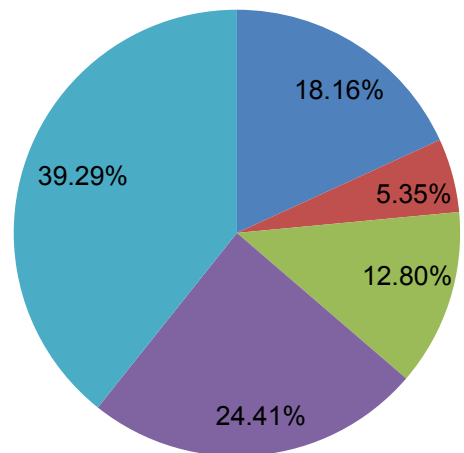

Supplement: Supplementary file 2 — 10.1186/s40709-016-0048-5 Length distribution of the ORFs predicted from the transcripts of six wild Nicotiana species. Length values are represented in amino acids. [file 40709_2016_48_MOESM2_ESM.pdf]

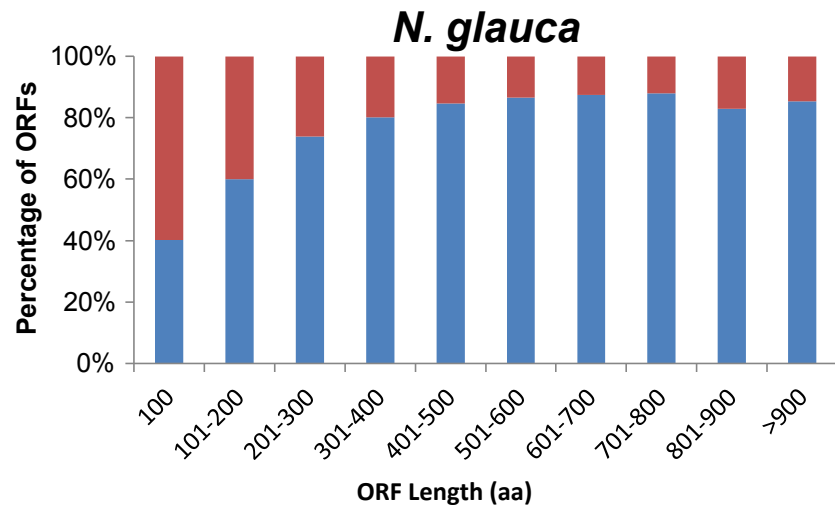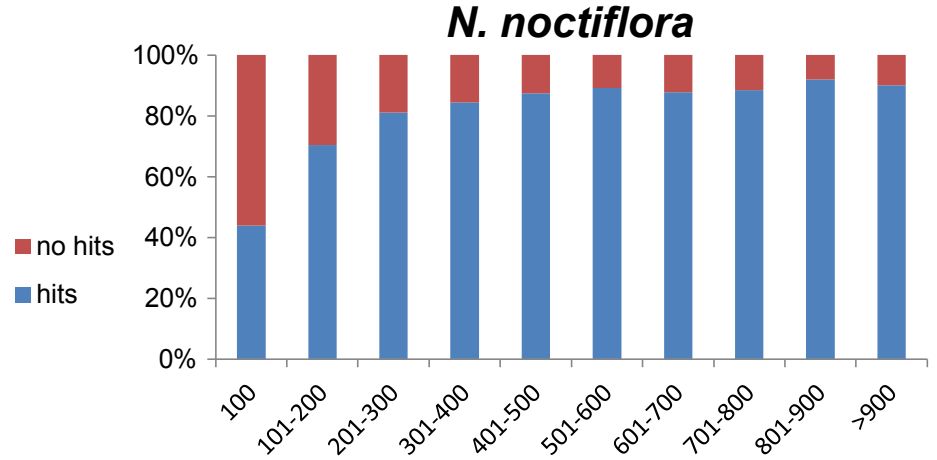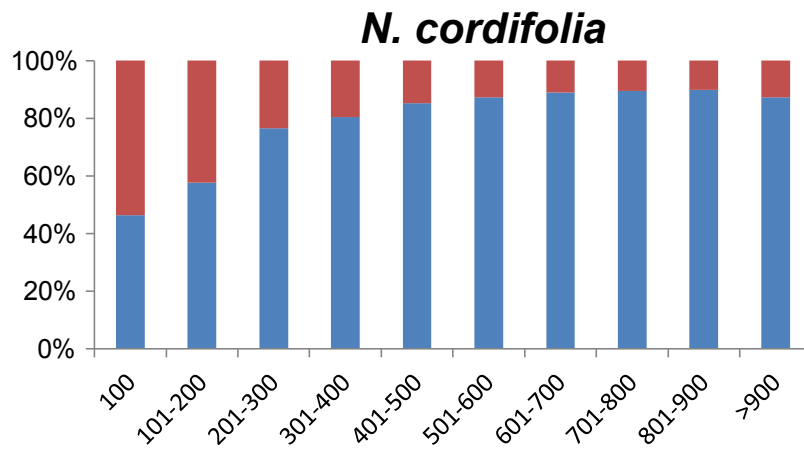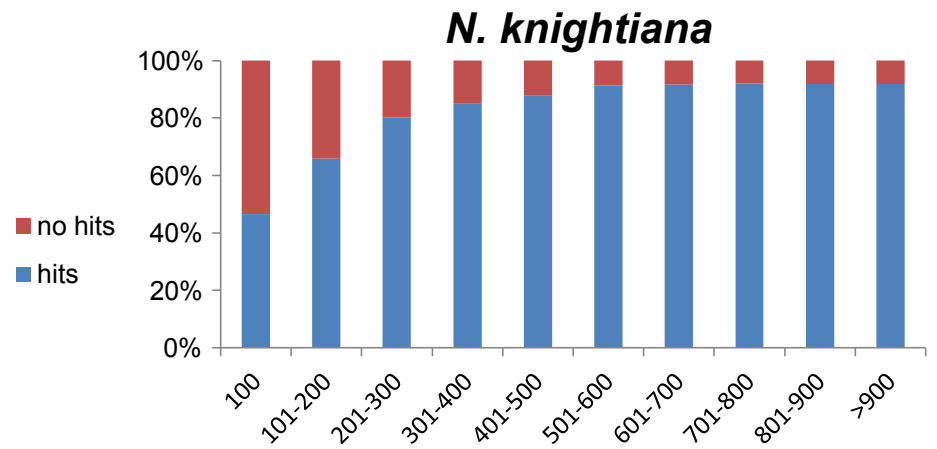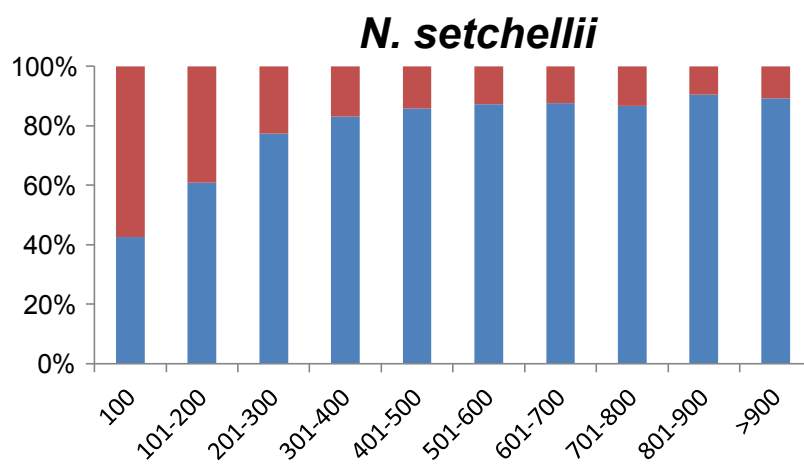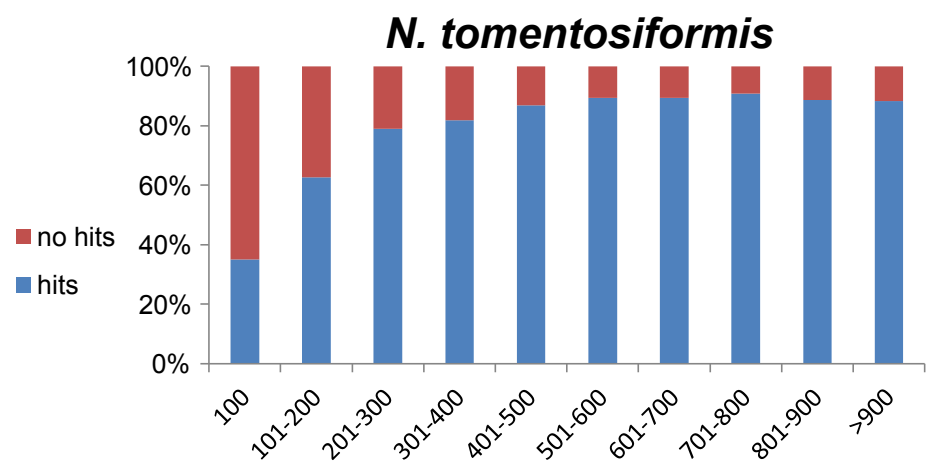

Supplement: Supplementary file 3 — 10.1186/s40709-016-0048-5 E-value distribution of the top BLAST hits for each ORF in the Swiss-Prot database. The cutoff e-value was set to 10−5. [file 40709_2016_48_MOESM3_ESM.pdf]

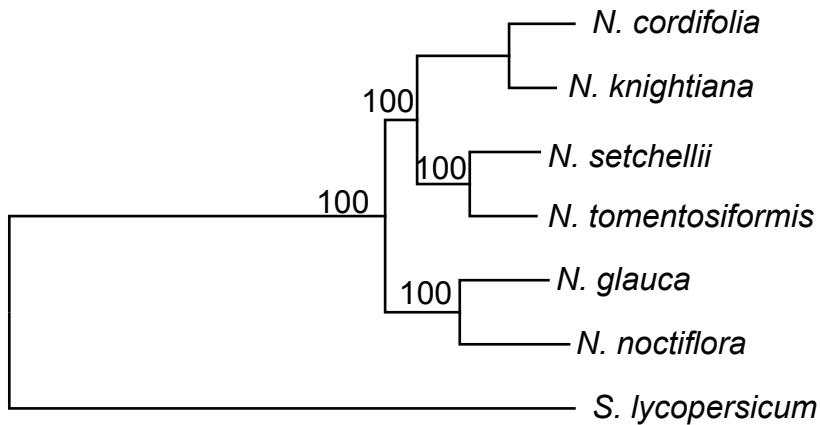

0.01

Supplement: Supplementary file 4 — 10.1186/s40709-016-0048-5 Comparison of ORF length between hit and no hit proteins in Swiss-Prot database. For N. glauca, N. noctiflora, N. cordifolia, N. knightiana, N. setchellii, N. tomentosiformis, longer ORFs were more likely to have BLASTp homologs in protein database. [file 40709_2016_48_MOESM4_ESM.pdf]
