# Supplementary material for: Sequencing and characterization of leaf transcriptomes of six diploid Nicotiana species
Source: J Biol Res (Thessalon). 2016 Apr 18;23:6. doi: 10.1186/s40709-016-0048-5 (PMC4835900; doi:10.1186/s40709-016-0048-5)
Supplement: Supplementary file 5 — 10.1186/s40709-016-0048-5 The expression levels of ORFs (FPKM) in the six Nicotiana species. [file 40709_2016_48_MOESM5_ESM.docx]

| Species | Clean data  (Gb) | No. transcript  (>100bp) | No. transcript  （>2000bp） | N50 | N90 |
| --- | --- | --- | --- | --- | --- |
| *N. glauca* | 23.98 | 182,046 | 30,676 | 2,013 | 418 |
| *N. noctiflora* | 20.83 | 146,188 | 22,434 | 1,837 | 408 |
| *N. cordifolia* | 10.11 | 134,519 | 26,789 | 2,079 | 495 |
| *N. knightiana* | 11.74 | 67,073 | 6,719 | 1,476 | 329 |
| *N. setchellii* | 9.68 | 102,935 | 15,645 | 1,801 | 413 |
| *N. tomentosiformis* | 13.49 | 117,640 | 17,750 | 1,834 | 397 |
